# Performance Differences in Elite National Basketball Association and Women’s National Basketball Association Players Based Upon Whether the Dominant or Non-dominant Achilles Tendon Was Ruptured

**DOI:** 10.7759/cureus.57423

**Published:** 2024-04-01

**Authors:** David Weinberg, Frances Shofer, Jason Pan

**Affiliations:** 1 Physical Medicine and Rehabilitation, University of Pennsylvania, Philadelphia, USA

**Keywords:** players, performance, wnba, nba, non-dominant limb, dominant limb, achilles tendon rupture

## Abstract

Background

National Basketball Association (NBA) and Women’s National Basketball Association (WNBA) players with Achilles tendon ruptures have previously been noted to have a significant decline in performance post-injury. There has been recent anecdotal evidence that elite players with dominant Achilles tendon ruptures may be able to return at a higher level of play post-rupture.

Objective

This study aimed to evaluate for any differences in performance in higher-performing NBA and WNBA players with dominant versus non-dominant Achilles tendon ruptures pre- and post-injury.

Methods

This study was conducted at the University of Pennsylvania, Department of Physical Medicine and Rehabilitation. NBA and WNBA players with an Achilles tendon rupture from 1990 to 2020 were identified. Only elite players, indicated by an average player efficiency rating (PER) of >15 in either of the three seasons pre/post-injury, were included. The average PER, offensive rating, defensive rating, and usage percentage were compared in the three seasons pre- and post-injury.

Results

Eighteen players met the inclusion criteria, and nine each were classified as dominant and non-dominant Achilles tendon ruptures based on their primary shooting hand. There was no significant difference between the dominant and non-dominant rupture groups in any outcomes pre-injury, including age. The non-dominant cohort had a significant decline in PER (20.04 vs. 14.16; p < 0.001) and offensive rating (110.33 vs. 101.56; p = 0.004) post-injury. There was no significant difference observed post-injury in defensive rating or usage percentage. The dominant cohort had no significant difference in any outcomes post-injury, returning to the same level of play as pre-injury. Despite no difference existing between the groups at baseline, the dominant group performed significantly better post-rupture with regard to PER (19.56 vs. 14.16; p < 0.001) and offensive rating (114.00 vs. 101.56; p < 0.001) versus the non-dominant group.

Conclusion

Elite NBA and WNBA players with dominant Achilles tendon ruptures had no change in performance post-injury, returning to the same level of production as pre-injury. Post-rupture, they demonstrated notably superior outcomes versus the non-dominant group with regard to PER and offensive rating. The non-dominant rupture group experienced the same decline in PER and offensive rating post-injury observed in previous studies. The data indicate that elite NBA and WNBA players with a dominant Achilles tendon rupture have a much more favorable recovery post-injury and are able to return to the same level of performance.

## Introduction

An Achilles tendon rupture has been regarded as one of the most detrimental injuries in professional basketball players [1-6]. Previous studies have found that 20-30% of National Basketball Association (NBA) players do not return to play following Achilles tendon rupture and experience a significant decline in performance following rupture [2-4]. Players also exhibited a decline in minutes and games played, coupled with notably shorter careers compared to age-matched controls. A comparable study conducted on Women’s National Basketball Association (WNBA) players revealed similar outcomes, with only 83% of players returning to play after rupture and experiencing diminished performance post-injury [5,6].

Despite the results of previous studies, there has been some anecdotal evidence that higher-performing NBA and WNBA players with dominant limb Achilles tendon ruptures have elevated performance post-injury, returning to similar levels of play as pre-injury [7,8]. However, no prior research has assessed how the rupture of either the dominant or non-dominant Achilles tendon affects player production. In general, there has been very limited data examining differences in the structure of the Achilles tendon in the dominant versus non-dominant limb, regardless of whether the tendon was ruptured. Past studies in athletes have noted that the dominant Achilles tendon at baseline may be thicker with an increased cross-sectional area in comparison to the non-dominant tendon [9-11].

Moreover, prior studies did not focus only on higher-performing players, instead encompassing all NBA or all WNBA players with an Achilles tendon rupture [2-6]. The average career of NBA players is approximately 4.5 years, so it is difficult to assess whether an Achilles tendon rupture was the inciting etiology for the end of a player’s career or just an accelerant to their natural career progression. It has been observed that higher-performing or “elite” professional basketball players have longer playing careers compared to lesser-performing players [12]. It is hypothesized that elite NBA and WNBA players may be able to return at a higher level of play following an Achilles tendon rupture compared to their peers. Therefore, the objective was to analyze only higher-performing NBA and WNBA players, evaluating for the differences in performance in players with dominant versus non-dominant Achilles tendon ruptures.

## Materials and methods

Study design

This study was conducted at the University of Pennsylvania, Department of Physical Medicine and Rehabilitation. It was deemed exempt by the University of Pennsylvania Institutional Review Board (IRB). A retrospective cohort study of NBA and WNBA players with documented Achilles tendon ruptures from 1990 to 2020 was performed. As done in prior studies, players were identified through publicly accessible data available via various methodologies, such as news articles, media reports, team sites, and NBA.com or WNBA.com. Players were then categorized into dominant or non-dominant Achilles tendon ruptures based on their primary shooting hand and which limb the rupture occurred in. A player who shoots primarily with their right hand, for example, was considered right-leg dominant, and vice versa if the player was left-handed. Each player’s primary shooting hand was verified using basketball-reference.com. In order to account for “elite” or higher-performing NBA or WNBA players, only players with an average player efficiency rating (PER) of greater than 15 in either the three seasons prior to or after Achilles tendon rupture were included in the analysis. PER is a performance metric that, through an established formula, incorporates a multitude of offensive and defensive statistical outcomes into a single number. The rating is adjusted on a per-minute basis and incorporates a team’s pace of play. The league average is consistently set to 15, so players with a PER greater than 15 would be deemed superior to the league average. 

Statistical performance for each player was averaged in three seasons pre- and post-injury using publicly available data on basketball-reference.com. The difference in performance for each outcome was then compared pre- to post-injury for each player, so each player served as their own control. To obtain a statistical baseline, players were only included if they had played at least three seasons prior to rupture and at least two seasons following rupture. If a player had only played two seasons following injury, the data were then averaged over only two seasons. The season in which the player ruptured their Achilles tendon was considered as one of the three seasons pre-injury, as all ruptures were season-ending injuries. To account for the variation in games played per season, a weighted average was utilized. For example, if a player played 100 games total over three seasons and played 30 games in season one, then that season would count toward 0.3 or 30% of the overall weight toward the average of that performance outcome. This was performed to account for the potential variation in games played per season. 

The primary outcome measurements for this study were PER, offensive rating, defensive rating, and usage percentage. As described previously, PER incorporates multiple statistical measurements into one number, attempting to measure a player’s true overall performance. Offensive rating specifically measures the number of points produced by a player per 100 possessions. Thus, a higher offensive rating indicates more points generated for the team by that individual player. Defensive rating measures the number of points allowed to the opposition by that individual player per 100 possessions. A lower defensive rating indicates fewer points allowed and would represent better performance. Usage percentage is an estimated percentage of the total team plays involving that player while they are in the game. A higher usage percentage would indicate that the player was more involved in the offense during their team’s possessions. 

Players were excluded if they were greater than 35 years old at the time of injury or if the specific limb side that was injured was unable to be verified through publicly accessed data. One player had switched primary shooting hands during their career and thus was excluded from the study.

Statistical analysis

To determine the differences in each performance outcome pre/post injury and between each rupture group, a two-way analysis of variance in repeated measures was performed. The rupture group (dominant/non-dominant) served as the fixed factor, and time (pre- and post-injury) served as the repeated measure. To adjust for multiple comparisons, post-hoc pairwise Tukey-Kramer tests were performed to reduce the likelihood of a Type I error. Summary statistics are presented as mean ± standard deviation for continuous data and frequencies and percentages for categorical data. A probability <0.05 was considered statistically significant. All analyses were performed using SAS statistical software (version 9.4, SAS Institute, Cary, NC).

## Results

Eighteen players who met the inclusion and exclusion criteria were identified and included in the study. Nine players were classified as dominant Achilles tendon ruptures, and nine players were classified as non-dominant Achilles tendon ruptures based on their primary shooting hand as previously described. Fifteen of the 18 players played at least three seasons following injury. The remaining three players played in only two seasons post-injury, with two of the three players still active in the league at the time of submission. Among the 18 players, 14 were in the NBA and four were in the WNBA. All players in the study had undergone surgical intervention of the Achilles tendon prior to returning to play. 

There was no significant difference in age at the time of rupture (28.21 vs. 29.64; p = 0.27) when comparing the dominant and non-dominant groups, respectively (Table 1). The average age for all players was 28.92 (range 24.67-34.67). At baseline pre-injury, there were no statistical differences between the dominant and non-dominant groups in any performance outcome: PER (p = 0.99), offensive rating (p = 0.76), defensive rating (p = 0.76), or usage percentage (p = 0.38). 

**Table 1:** Demographics of the dominant and non-dominant Achilles tendon rupture groups Demographic information between the two groups. Values are represented as mean age ± standard deviation at the time of Achilles tendon rupture and the number of players in the NBA and WNBA in each cohort.

Demographics	Dominant (N = 9)	Non-dominant (N = 9)	p-value
Age at rupture	28.21 ± 2.60	29.64 ± 2.70	0.27
Number of players in NBA (N)	N = 6	N = 8	N/A
Number of players in WNBA (N)	N = 3	N = 1	N/A

For the non-dominant Achilles tendon group, there was a decline in all four performance outcomes post-rupture. For PER, there was a 5.88-point decline from 20.04 pre-injury to 14.16 post-injury (p < 0.001; Table 2). Similarly, offensive rating declined from 110.33 to 101.56 (difference of -8.78; p = 0.004). Both defensive rating and usage percentage declined as well (as increased defensive rating indicates a decline in performance), but no statistically significant differences were noted (Table 2). 

**Table 2:** Performance outcomes in the non-dominant Achilles tendon rupture group pre- and post-injury Average performance outcome in the three seasons pre- and post-injury in the non-dominant Achilles tendon rupture group. Outcomes are represented as mean ± standard deviation. Bold p-values indicate statistical significance (p < 0.05).

Performance outcome	Pre-injury	Post-injury	Difference (post/pre)	p-value
PER	20.04 ± 3.33	14.16 ± 3.13	-5.88	<0.001
Offensive rating	110.33 ± 4.00	101.56 ± 4.25	-8.78	0.004
Defensive rating	105.78 ± 2.99	108.44 ± 4.50	2.67	0.078
Usage percentage	26.06 ± 6.38	22.98 ± 5.84	-3.08	0.058

Conversely, for the dominant Achilles tendon rupture group, there was no significant difference in PER, offensive rating, defensive rating, or usage percentage when comparing performance post- and pre-rupture (p > 0.38 for all; Table 3).

**Table 3:** Performance outcomes in the dominant Achilles tendon rupture group pre- and post-injury Average performance outcome in the three seasons pre- and post-injury in the dominant Achilles tendon rupture group. Outcomes are represented as mean ± standard deviation.

Performance outcome	Pre-injury	Post-injury	Difference (post/pre)	p-value
PER	20.03 ± 5.36	19.56 ± 5.10	-0.48	0.97
Offensive rating	112.44 ± 9.84	114.00 ± 11.75	1.56	0.89
Defensive rating	104.78 ± 8.35	106.44 ± 8.53	1.67	0.38
Usage percentage	24.23 ± 4.62	24.37 ± 5.80	0.14	0.99

When comparing the dominant and non-dominant groups, there were no differences pre-injury between the groups in any of the outcome measurements. Post-injury, the dominant group demonstrated significantly higher performance in PER in comparison to the non-dominant group (19.56 vs. 14.16; p < 0.001; Table 4). Similarly, there was significantly higher performance post-rupture in offensive rating compared to the non-dominant group (114.00 vs. 101.56; p < 0.001; Table 4). There was no significant difference between the groups with regard to defensive rating or usage percentage post-injury (Table 4).

**Table 4:** Post-injury performance in the dominant versus non-dominant rupture group Average performance outcomes in the three seasons post-injury in the dominant and non-dominant Achilles tendon rupture group. Outcomes are represented as mean ± standard deviation. A bold p-value indicates statistical significance (p < 0.05).

Statistical category	Dominant	Non-dominant	p-value
PER	19.56 ± 5.10	14.16 ± 3.13	<0.001
Offensive rating	114.00 ± 11.75	101.56 ± 4.25	<0.001
Defensive rating	106.44 ± 8.53	108.44 ± 4.50	0.24
Usage percentage	24.37 ± 5.80	22.98 ± 5.84	0.60

## Discussion

Achilles tendon ruptures have been considered one of the most detrimental injuries in professional basketball players, overall leading to shortened careers and significantly decreased performance following injury [1-6]. Earlier studies examined Achilles tendon ruptures in either all NBA or WNBA players, without specifically focusing on elite or higher-performing players based on their PER [2-6]. Prior research also did not explore the potential disparities in performance in NBA or WNBA players with dominant versus non-dominant Achilles tendon ruptures. Therefore, this is the first known study to compare performance outcomes in professional basketball players based on limb dominance and which Achilles tendon was ruptured.

The results indicate that elite NBA and WNBA players with dominant limb Achilles tendon ruptures had no significant change in performance post-injury in any outcome measurements. From a performance standpoint, players with dominant limb ruptures were able to return to the same level of play as pre-injury with regards to PER, offensive rating, and defensive rating. Some players in the dominant cohort actually demonstrated improvement in PER and offensive rating after injury, although overall there was no significant difference amongst the group. Conversely, elite NBA and WNBA players with non-dominant Achilles tendon ruptures experienced a marked decline in both PER and offensive rating post-rupture, but no significant change was observed with regard to defensive rating. When comparing the groups, there was no significant difference between the dominant and non-dominant groups pre-rupture in any outcome or age at the time of rupture. Post-injury, however, the dominant cohort demonstrated significantly superior performance offensively compared to the non-dominant cohort, returning to the same elite level of production as pre-injury. Therefore, there is a significant difference in performance following an Achilles tendon rupture based on whether an elite player's dominant or non-dominant tendon was ruptured. These findings suggest that higher-performing NBA and WNBA players with a dominant Achilles tendon rupture have a much more favorable prospect for recovery post-injury. 

In addition, there was no significant change in the usage percentage post-injury for both groups, suggesting that the players remained involved in the same amount of their team’s offensive plays after injury. Despite no difference in their overall involvement on offense, players with non-dominant ruptures still experienced notably diminished production offensively.

The reason for the superior outcomes observed in the dominant Achilles tendon rupture group post-injury is not fully understood. One possible explanation is that in basketball, athletes will utilize their non-dominant leg to propel themselves off the ground when attempting a lay-up or dunk. When beginning their initial acceleration toward the dominant side of the basket, players will initiate the push-off with their non-dominant leg. For instance, a right-handed player will leap off their left leg during a dunk and initiate acceleration to the right side of the basket by pushing off with their left leg. Although this may account for a small difference in performance, professional basketball players rely on a complex array of motions and need to utilize both limbs and sides of the basket, so the actual impact is unclear. 

In general, there has been limited data investigating the effect of limb dominance on the Achilles tendon, irrespective of whether the tendon was ruptured or not. Of the limited studies, a retrospective study on healthy professional badminton players found that the dominant Achilles tendon demonstrated greater thickness, cross-sectional area, and width compared to the non-dominant tendon [11]. In a study on physically active male adults who were not involved in sports, it was observed that the Achilles tendon of the dominant limb had a higher Young’s modulus (indicating increased resistance to elastic deformation under load) and length compared to the non-dominant tendon [10]. A study on long-jump collegiate athletes found that the dominant Achilles tendon had increased stiffness and a greater Young’s modulus than the non-dominant tendon [9]. Prior research has noted that there is a correlation between increased Achilles tendon thickness and cross-sectional area with increased acceleration and velocity in athletes [13,14]. It is possible that the dominant Achilles tendon is more likely to return closer to its biomechanical baseline post-injury, given the potential increase in thickness, Young’s modulus, and cross-sectional area pre-injury. This would then account for the superior performance observed post-rupture compared to the non-dominant group. No study has specifically assessed for this in individuals with Achilles tendon ruptures, however. Conversely, a study performed on healthy amateur basketball players found a greater stiffness in the Achilles tendon bilaterally compared to non-athletes but observed no significant difference in stiffness between the dominant and non-dominant sides [15]. Therefore, more research is needed to further elucidate the differences in Achilles tendon structure and function between the dominant and non-dominant limb post-rupture.

In addition, one of the major complications following an Achilles tendon rupture is the increase in gastrocnemius and soleus muscle atrophy post-injury. Previous studies have found a notable increase in atrophy and fatty tissue infiltration in the gastrocnemius and the soleus muscles on the rupture side following Achilles tendon tear, resulting in decreased functional outcome scores [16-18]. These pathophysiological changes are believed to be a part of the reason for the decline in performance in athletes following injury [16,18]. It is unknown whether athletes with dominant limb ruptures are less susceptible to triceps surae atrophy and fatty infiltration compared to non-dominant ruptures, so additional investigations are needed. 

This study specifically evaluated only higher-performing NBA or WNBA players or players with an average PER of greater than 15. Although the average NBA career is only 4.5 years, previous research has noted a strong correlation between elevated player performance and career longevity [12]. Thus, it was hypothesized prior to the study that higher-performing players might be able to return to a similar level of play post-rupture, given their projected career longevity. However, this was only observed in players who ruptured their dominant Achilles tendon. It is unknown if the results of prior studies, which included all NBA or WNBA players, would be different if subgroup analyses were conducted based on whether the dominant or non-dominant tendon was ruptured. It is also unclear if these results are applicable to other professional sports where athletes have experienced a decline in performance following an Achilles tendon rupture [19-21].

The main objective for future studies would be to elucidate the various factors behind the performance disparities observed between the dominant and non-dominant groups. At this time, there are no known variations in rehabilitation protocols based on whether the dominant or non-dominant Achilles tendon is ruptured. Depending upon the results of future research, it may be possible that different rehab protocols should be utilized to mitigate potential differences in functionality. It is possible that which Achilles tendon, whether the dominant or non-dominant, was ruptured may help serve as a prognostic indicator for performance post-injury in elite professional basketball players. Ultimately, additional studies are needed and warranted on this subject. 

There were a few limitations to the study. The sample size was small (n = 18), consisting predominantly of NBA (n = 14) players compared to WNBA (n = 4) players. Due to the small sample size, there was no subgroup analysis to compare NBA versus WNBA players. There was, however, an equal distribution between the two groups: dominant (n = 9) and non-dominant (n = 9). It is also possible that no statistically significant differences were seen in the usage percentage or defensive rating in the non-dominant group because the study was underpowered. As time progresses, the sample size is anticipated to increase as more elite players return following an Achilles tendon rupture, so this may ultimately change. Although the methods of obtaining injury and outcome data were similar to prior studies, there is no centralized NBA or WNBA database for injuries. It is likely that there were some variations in the rehabilitation protocol among the athletes studied. Unfortunately, the specific rehabilitation protocol each athlete underwent post-injury is not publicly available. Each player was also on a different team with a different medical staff at the time of injury. Given that rehabilitation protocols do not, to our knowledge, vary based on whether the dominant or non-dominant Achilles tendon was ruptured, it is unlikely that there would be significant variations in the rehabilitation plan between the two cohorts that impacted the results. It is also likely that there were variations in the surgical intervention performed among the athletes studied.

## Conclusions

Elite NBA and WNBA players with dominant Achilles tendon ruptures had no change in performance following rupture, returning to the same level of play as pre-injury. Post-injury, they demonstrated notably superior outcomes versus the non-dominant group with regard to PER and offensive rating. The non-dominant rupture group experienced the same decline in PER and offensive rating post-injury as observed in previous studies. The data indicate that elite NBA and WNBA players with a dominant Achilles tendon rupture have a much more favorable recovery post-injury and are able to return to the same level of performance. Further research on this subject is warranted.
